# Family carers’ involvement strategies in response to sub-optimal health services to older adults living with dementia – a qualitative study

**DOI:** 10.1186/s12877-020-01663-z

**Published:** 2020-08-17

**Authors:** Kristin Häikiö, Mette Sagbakken, Jorun Rugkåsa

**Affiliations:** 1 HØKH – Health Services Research Unit, Akershus University Hospital, Sykehusveien 27, 1478 Lørenskog, Norway; 2 Oslo Metropolitan University, St.Olavs plass, 0130 Oslo, Norway; 3 Centre for Care Research, University of South-Eastern Norway, Kjølnes ring 56, 3901 Porsgrunn, Norway

**Keywords:** Dementia, Caregivers, Health services for the aged, Health literacy, Social capital, Community care

## Abstract

**Background:**

While dementia policy strategies emphasize the importance of partnerships between families and formal carers to provide tailored care and effectively allocate community resources, family carers often feel left out or excluded. Poor communication has been identified as one reason for the lack of good partnerships. Few studies have investigated how family carers seek to involve themselves when they experience sub-optimal services, and how their strategies may depend on different considerations and personal abilities.

**Methods:**

Qualitative in-depth interviews were conducted with 23 family carers to explore their experiences with, perspectives on, contributions to, and interactions with healthcare services provided to older adults living with dementia. To capture nuances and variations, a semi-structured interview guide was used. Interviews were audio-recorded and transcribed verbatim. A four-step analysis of the transcripts was conducted, informed by hermeneutic and phenomenological methodology.

**Results:**

Two main involvement strategies were identified: 1) being “the hub in the wheel” and 2) getting the wheel rolling. The first strategy was used to support and complement health services, while the second was used to add momentum and leverage to arguments or processes. The two main strategies were used differently among participants, in part due to differences in personal resources and the ability to utilize these, but also in light of family carers’ weighing conflicting concerns and perceived costs and benefits.

**Conclusions:**

Awareness and acknowledgment of family carers’ strategies, personal resources, and considerations may help policymakers and healthcare personnel when they build or maintain good partnerships together with family carers. A better understanding of family carers’ own perspectives on carer involvement is a necessary precursor to developing good care partnerships.

## Background

The number of people living with dementia is increasing worldwide; consequently, dementia care represents a challenge to future health services. Family carers are contributing significantly to the care of older adults living with dementia [1], in terms of personal care, supporting daily activities, and interacting with healthcare personnel [2–5]. Their role is described in international and Norwegian health policy documents as a means of enhancing the quality of care, utilizing potential care recourses in both formal (services) and informal (family) sectors, and providing care tailored to individual needs [6, 7]. In many countries, dementia strategies [6, 8–10], healthcare reforms for senior citizens [7, 11], and action plans to support family carers [12, 13] emphasize that partnerships between healthcare personnel and family carers are needed to achieve high quality and sustainable care. According to the World Health Organization (WHO), partnerships in care should be based on trust, equality, mutual understanding, shared goals, and shared accountability [14]. This is reflected in policy documents, such as the UK strategy for family carers, which emphasizes that healthcare personnel should consider family carers as partners in care and recognize their unique expertise [13, 15].

The Norwegian national dementia strategy [10] highlights how family carer involvement can help to prolong the period of independent living for those living with dementia. Like other countries, Norway has developed guidelines for how services are expected to involve family carers [8, 16]. “Involvement” is described in various ways, but it usually entails information sharing, reciprocal discussions, and having a say in decision making throughout the care pathway [17, 18]. Even though many people living with dementia are capable of participating in decisions about their care [19], carer involvement is recognized as increasing in importance as care recipients’ symptoms progress [20].

Despite the good intentions of policies, research has identified substantial differences in family carers’ personal experiences of involvement in dementia care [21, 22] In the UK and in Norway, some studies report that many family carers are satisfied with the ways they are involved and supported in their role [23, 24] other studies, in contrast, find that many family carers experience that they are not involved, despite their important insights into the situation of the person living with dementia [6, 21, 25]. Some carers experience being actively excluded by healthcare personnel, which is sometimes explained by patient confidentiality [26]. Obstacles to well-functioning partnerships may include poor communication and a lack of feedback from relatives or staff [27]. In many countries, advocacy groups have emerged to support family carers in interactions with services [28].

The ways in which family carers and healthcare personnel interact are based on long traditions of hierarchical approaches to working together [29], which are sustained through education [30] and role expectations [3]. This is often seen to result in power imbalances between family carers and healthcare personnel, which create barriers to “real” or equal partnerships [31, 32]. SeParticipant 2 l concepts have been developed to help understand these power imbalances and how people are differently equipped to address them. Social [33] and cultural capital [34] refer to personal resources in terms of connections or knowledge that can impact on such interaction. The concept of Health literacy (HL) is linked to such personal resources in the form of knowledge, motivation, and competency in terms of accessing, understanding, appraising, and applying information about health and healthcare systems [35]. HL can be understood as a particular level of skills or competencies to capitalize on knowledge relevant to the health field. HL has become increasingly important in public health and health care over the recent decades, and it contextualizes people’s approaches to health services in order to develop an understanding of what shapes these approaches [35].

As described in the literature, family carers can perceive that services sometimes provide insufficient or sub-standard care [36–38], and many studies have explored family carers’ experiences in such situations [15, 38–40]. There is a dearth of studies, however, that have investigated what family carers do in response to what they perceive as insufficient care. This is the focus of our study. Rather than discussing how services involve—or fail to involve—family carers, we investigate how family carers seek to involve themselves in formal service provision when they perceive that services, for various reasons, are not providing optimal care to their relative living with dementia. We believe this perspective is important to understanding family carers’ perspectives on carer involvement, which is a necessary precursor to developing good care partnerships and improved care pathways for families affected by dementia.

## Method

### The Norwegian service context

Norway offers a tax-based public health care service [41]. Primary dementia care is the responsibility of the approximately 350 municipalities, and typically includes general practitioners (GPs,) home care, nursing homes, and respite care. Respite care can be residential or day-time facilities. Most municipalities also have dementia teams or dementia coordinators, who support, plan, and coordinate care. Four Regional Health Authorities provide specialist dementia care through hospitals, memory clinics, or geriatric outpatient clinics [42]. In accordance with the principle of the lowest level of effective care [43, 44], people living with dementia receive most services from primary care providers and while living at home. National Guidelines for how services should involve family carers were updated and reissued in 2017 [16].

### Design

Eligible participants included persons who provided unpaid support to a family member, friend, or neighbor who was aged 65 years or older and who received health services due to suspected or diagnosed dementia. Participation was voluntary and written consent was given from each participant prior to interview.

To achieve maximum variation [45–47] in the experience of caregiving, we actively tried to balance the sample in terms of gender, relationship to the care recipient, whether participants lived in rural or urban areas, or whether they were born in Norway or abroad.

We constructed a semi-structured interview-guide, based on previous research on family carers’ experiences with health services, including services to older people living with dementia. A draft guide was refined in collaboration with the research group’s user-panel, consisting of non-researchers with diverse experiences of different health services. It was piloted with one of the panel members and was found to work well according to the purpose. The interview-guide was finally informed by previous research about family carers and dementia care [5, 25, 48–52]. Given the hermeneutic and phenomenological approach, the topic guide consisted of open questions about subjective experiences. Topics surrounded family carers’ experiences of their contributions to and interactions with health services, and different views on service integration and quality. Examples of questions include: “How did you come in contact with health services?”, “Can you tell me more about the health services he/she is using?”, “Can you tell me about a good/bad interaction with the health services?” The guide ensured that we discussed the same topics in all interviews while capturing nuances and encouraging reflection [53]. It was applied in a flexible and exploratory manner that allowed the interviewer to follow up on points raised and allowed participants to bring up topics or issues relevant to them.

### Sampling and data generation

We used purposive sampling using three strategies: 1) asking public healthcare personnel—such as dementia coordinators and staff in home care, hospital, and nursing homes—to recruit on our behalf by distributing a study information sheet. This resulted in 20 family carers volunteering to participate; 2) publishing an open invitation through Facebook, which resulted in 2 participants; and 3) using snowball sampling [54] through participants already interviewed, which resulted in 4 participants. A total of 26 family carers volunteered. Of these, three cancelled: two of them due to illness and one without giving a reason. We do not have information of how many participants who refused to participate or any characteristics of invited non-participators.

After the first 11 interviews, we sought to balance the sample by focusing recruitment activities towards male family carers and family carers born abroad. A saturation point was discussed after 16 interviews, but as these all came from the southern part of the country, included an additional 5 participants from the north of the country.

Interviews were conducted between June and October 2017. The interviewer (KH), who had some previous experience with qualitative interviews, presented herself as a PhD-candidate with extensive clinical nursing experience from emergency care, but not from dementia care.

Participants chose the time and place for the interviews; most of them were conducted in participants’ homes or at the researcher’s office. The interviews were carried out as a private conversation between KH and the participant, and all interviews were held in Norwegian. Participants were informed about their right to withdraw from the interview in whole or in part; none of them did. It was emphasized that data analysis and reporting would maintain confidentiality. The interviews lasted approximately 1.5 h and were digitally audio-recorded. No field-notes were made, but the interview was transcribed the same day or within a few days, usually before the next interview. Non-verbal language was written as comments in the transcripts when it was necessary to explain use of irony or emotions not captured by the text. Transcripts were not returned to participants for comments.

Participants seemed motivated to tell their story, and the interviews provided rich descriptions of their perspectives. Often, participants started to talk about their experiences before the first question was asked, and the researcher then guided the conversation to a relevant section of the topic guide. Participants were interviewed once.

As shown in Table 1, our final sample of 23 participants varied with regard to background characteristics. Some cared for relatives living in a nursing home, but most cared for relatives living at home. The conditions of the persons they cared for (symptoms, whether or not dementia was formally diagnosed, functioning level, and illness progression) also varied, as did kinship relations and living arrangements. Participants collectively had experiences of interacting with a wide range of services.

**Table 1:** Characteristics of the sample

Characteristics	*n*=23	
Gender, n (%):		
Female:		17 (74)
Male:		6 (26)
Age, years min-max (median) :		44-83 (62)
Relationship, n (%):		
Spouse		12 (52)
Adult children		9 (39)
Adult siblings		2 (9)
Geography, n (%):	Urban areas, *n*=14 (61)	Rural areas, *n*=9 (39)
North of Norway, n=6	0	6
East of Norway, n=17	14	3
Living arrangements, n (%):		
Shared household with the person with dementia		11 (48)
Not sharing household with the person with dementia		6 (26)
Care recipient lived in nursing home		6 (26)

While we achieved a broad variation of experiences in the sample, it is likely that it consists of family carers who were eager to share their experiences. The sample might lack the perspectives of family carers who are less engaged in caring or for various reasons did not want to share their personal caring experiences.

### Data analysis

In this paper we report on the second strand of analysis from the data generated from interviews guided by the semi-structured interview guide and previous work as reported previously [55]. Our previous work identified how communication problems inadvertently can result in health services not meeting the needs of people living with dementia [55]. We therefore focused the present analysis on how participants endeavored to involve themselves in formal service provision when they felt that services did not provide optimal care, and identified various strategies. The analysis is informed by hermeneutic and phenomenological methodology [56, 57], which focuses on understanding experiences from subjective viewpoints and developing insight through cycles of analyses and reflection [53].

We conducted a four-step analysis, combining different qualitative techniques. Step 1 consisted of transcribing all data and obtaining an overview [58]. The transcripts were made in Norwegian and only quotations used in the article were translated to English. Step 2 consisted of an interim overview analysis exploring different perspectives, interpreting the data in light former research, empirics, and researchers preunderstanding [59–61]. Step 3 involved detailed inductive description through line-by-line coding of all text in the computer software NVIVO, version 11. This resulted in 1383 inductive codes, organized in 51 categories. In Step 4, we combined the interpretations and preliminary findings from Step 2 with the codes from Step 3 and connected codes with themes. All themes were thus derived from the data. These steps are described more extensively elsewhere [55]. Data analysis was led by KH, in close collaboration with JR and MS who both are social scientists with many years’ experience of qualitative research.

In the present analysis, for the detailed analysis in Step 4 we applied codes related to participants’ discussion surrounding how they sought to involve themselves in service. We categorized the various approaches described into two main themes that we call *involvement strategies*: 1) being “the hub in the wheel”, and 2) getting the wheel rolling, each with seParticipant 2 l subthemes. The categorizations emerged in discussions among the authors, and through feedback from the wider research group (but not by participants). Below, we present the use of the two main strategies and the specific approaches constituting each strategy. We also show how different approaches are sometimes weighed against each other and against considerations of potential costs and benefits. We use quotes from the transcripts to illustrate our interpretations [62], and pseudonyms are used to ensure participants’ privacy. Unless otherwise specified, the quotations represent common views in the sample. Results were not discussed with participants.

## Results

### Being “the hub in the wheel”: a supportive and complementary strategy

Participants interacted with a variety of service providers, and they frequently recognized discontinuities between these providers and/or between the providers and families. To contribute to more seamless care, participants described different approaches used to support and complement services: 1) rectifying incomplete information flows, 2) connecting disjointed services, and 3) filling care gaps.

#### Rectifying incomplete information flows between families and services

Many participants had experienced incomplete information flows between themselves, the person living with dementia, and healthcare personnel. They saw the role of family carers very clearly as providing accurate and up-to-date information so that services could be tailored to the needs of the person living with dementia. Many participants experienced that their relative trivialized dementia symptoms or were unable to pass on accurate/complete information to healthcare personnel. Therefore, many participants accompanied their relative to all appointments with GPs, hospitals, other healthcare personnel, or the pharmacy. Participant 1, who lived with a spouse with dementia, talked about how they rectified faulty information flows between the family and service providers:

*I always go with* [care-recipient] *to check-ups, because I realized that I had to, otherwise I wouldn’t hear anything. Because* [care recipient] *just said, “No, there wasn’t anything in particular.” And also I had to tell* [healthcare personnel] *how* [care recipient] *functions.* [Care recipient] *trivializes that whenever he/she is at check-ups. So I’ve been with* [care recipient] *everywhere.* (Participant 1, aged 70–80, caring for a spouse)

Many also wrote letters to the GP, home care services, the municipal commissioning office, or to nursing home staff to ensure these professionals were fully informed about their relative’s situation and needs.

Some participants were concerned that sometimes, when they provided information to staff about needs that their relative was unable to express, it was not always passed on between shifts or between different providers. Many participants tried in different ways to rectify this. Participant 2 had talked to staff at the care recipient's nursing home several times about how their  mother should be dressed to keep warm. When the situation did not improve, the family carer wrote down instructions:

*She gets cold very easily. You know, old people sit still a lot and they easily get cold…But I wrote* [a note to staff] *that she needs to wear a long-sleevedt-shirt and a blouse and her cardigan and a kerchief.* (Participant 2, aged 40–50, caring for a parent)

Participant 2 thus attempted to correct what they saw as insufficiently tailored care by personally making the information available for staff across shifts.

Participant 3 explained that their mother, who suffered from Chronic Obstructive Pulmonary Disease (COPD) in addition to dementia, was unable to provide accurate information to ambulance personnel when an exacerbation required hospital admission. Therefore, Participant 3 made sure that the most recent discharge note was left visible:

*I always leave the discharge note on her table where she also has her ventilator and everything is there. So if she struggles to breathe and presses the button* [on her alarm] *and the ambulance comes and she says “oh, my back is so sore,”* [but] *it isn’t that, that’s not why she calls. So then they at least have the discharge note and they have all the information about her diagnosis and my number and the name of her GP.* (Participant 3, aged 50–60, caring for a parent)

Participant 3, like many other participants, portrayed their self as central to rectifying disrupted information flows because of their relatives’ lack of capacity or ability to express themselves.

#### Connecting disjointed services

Several participants discussed how the different services their relative was in receipt of, often were disjointed, and how they involved themselves to coordinate them. Participant 1 explained that, contrary to hospital policy, discharge notes were not always provided to other services involved in their spouse’s care, and Participant 1 would take steps to make sure that the notes were distributed appropriately:

*Participant 1: And we’ve asked* [hospital staff] *that the specialist in town* [private specialist in internal medicine] *gets the discharge note. So that, but this last time the GP hadn’t received it. Sometimes it slips, but we’ve started asking wherever we are, that discharge notes are sent at least to the GP and the internist.*

*KH: So you keep an eye on where it’s sent, to make it work?*

*Participant 1: Yes (laughs). We’ve discovered that we need to do that. Because at the beginning it wasn’t sent automatically.* [care recipient] *has been at another hospital too, but the two hospitals don’t send* [information] *to each other. Because of confidentiality and stuff.* (Participant 1, aged 70–80, caring for a spouse)

Several participants had similar experiences about the lack of services sharing information with each other. Participant 3 found that they was often the one informing one service about the activities of another, and even about where their mother was at any given time:

*When my mother is discharged* [from the hospital]*, there is an automatic message to the home care that she’s returning home now. But this has been missed a few times…So like 2-3 weeks passed. And then I called the ones who are going to help her shower, because that’s, you know, another unit, and they said, “yeah, but we thought she was at the hospital still. We haven’t been told she’s back.” And then I said, “Yes, she is.”* (Participant 3, aged 50–60, caring for a parent)

Several participants said that one of their most important roles was to obtain a full overview and to connect services when needed: Participant 4 described this as being the “hub in the wheel”. This often also included changing appointments that conflicted, or letting home services know when they needed to finish their tasks for the day so that the care recipient could make it to other healthcare appointments on time.

#### Filling care gaps

Participants tried to complement and support service provision by doing tasks “here and there” when they saw it was needed. These were tasks that healthcare personnel forgot or did not have the time to do, or aspects of care that were considered by family carers to be important to the care recipient but were not necessarily prioritized by healthcare personnel. For example, Participant 5 explained that the nursing home staff did not have time to sit with the spouse [care recipient] during meal times, and as a result, the care recipient ate poorly. Participant 5 therefore visited the spouse daily to sit down with the spouse to eat and drink.

In many cases, participants’ care recipient suffered from additional illnesses. Healthcare personnel involved in treating these illnesses did not always take into account their dementia related needs. Consequently, participants would often deem it necessary to help out so that their care recipient would, for example, show up at the right place and do what was required during assessments. Participant 4 explained how they provided such support when the spouse [care recipient] underwent minor surgery:

*So* [care recipient] *was called in for an operation* [at the hospital]…*And then* [care recipient] *went into a cubicle.* [Care recipient] *supposed to take off all clothes except his/her underwear, and valuables and clothes are to be put in a cupboard. And where is the cupboard?* [Care recipient] *had perhaps just left all those things* [out]*. Because there was no one there to help. There was a lady there to hand out these clothes, right, but there was no one there to help, you know. Because* [care recipient] *looks well, there isn’t an “A”* [indicating Alzheimer's disease] *on the forehead*. (Participant 4, aged 60–70, caring for a spouse)

Participants also gave examples of how they made sure the correct equipment was available. Participant 3, whose mother lived alone in her own apartment, said that their mother was once at an intermediate unit for observation after hospital discharge and before returning home, and she had been put in a wheelchair instead of given a walker. She was also left without her ventilator. Participant 3 describes how they rectified this:

*She uses a walker…And the first thing I see when I get in was that the nurse said, “Your mother is sitting over there in the corridor, and we managed to get a wheelchair from another patient when she told us she was paralyzed from the waist down.” And she is not paralyzed from the waist down. So they had not read the discharge note…“I don’t know where I am,”* [care recipient] *says, “I don’t know which room I live in.” So I found that out and got her in there and, and I had brought her walker in the car, and they didn’t have a ventilator either, so I had to go to her apartment and collect that.* (Participant 3, aged 50–60, caring for a parent)

Care gaps were filled in different ways in different situations, but this was commonly described as a way of tailoring care to personal needs and improving quality of care.

### Getting the wheel rolling: an assertive strategy

Participants described situations in which they deemed it necessary to engage in more assertive approaches, in order to add leverage or pressure to arguments or processes, so as to ensure adequate care quality. These included: 1) keeping healthcare personnel on alert, 2) using relationships as leverage, and 3) filing official complaints.

#### Keeping healthcare personnel on alert

Several participants said that in situations where they were concerned about the quality of care, they kept a closer eye on services/providers and made sure the healthcare personnel were aware of this. Participant 2, who cared for their mother in the local nursing home, explained that they had told the nursing home staff that they believed her mother’s frequent urinary tract infections were a result of her sitting for prolonged periods in wet diapers. Convinced that the staff did not act satisfactorily on this information, Participant 2 began to visit the care recipient at unpredictable hours—sometimes in the morning, sometimes in the evening, and sometimes in the middle of the day, to keep an eye on things. Participant 6 believed that the quality of care often depended on the skills and attitudes of individual healthcare personnel, and would confront them if they thought they were negligent or careless:

*And some of them* [the staff] *are, like: “I’ll sit down and check my phone and Facebook and that”…Then I say, “what are you doing sitting here fiddling with private things rather than sitting down to talk to those who live here, or simply doing your job?”* (Participant 6, aged 40–50, caring for a parent)

Participant 7 would also confront staff when they thought it was necessary. Parthicpant 7 discussed how they had pointed out to the staff at the nursing home that locking the doors was unlawful and potentially dangerous to residents, and made explicit reference to their own expertise as a specialist nurse:

*I asked* [staff at the supported housing]: *“Why do you lock the doors so that he can’t go in there? Are you not aware that you’re not allowed to lock the door? There has to be a legal order.” I said: “That’s not legal. You need to keep yourselves updated on laws and regulations! I am a Specialist Nurse, so I know these systems.”* (Participant 7, aged 50–60, caring for a parent)

It was a common experience that participants sometimes needed to be involved in order to keep healthcare personnel on alert and ensure adequate quality of care. Participant 7 used their professional expertise to do so. Participant 7 made it clear that obtaining knowledge was crucial for family carers so that they could be proactive and not “just having to accept things”.

#### Using relationships to add leverage

Participants discussed how they sometimes used social relationships to add pressure to situations where they disagreed with the service provider’s approach. Participant 8 had initially been unsuccessful when writing to healthcare services to get respite care for the spouse. Participant 8 said that they would not have been able to make headway had it not been for their own daughter’s who helped in arguing their case:

*But I have a resourceful family around me…It’s my eldest daughter who’s written letters and things. So the first letters we got back* [from respite services] *said that* [care recipient] *has a spouse who is at home, and children and grandchildren. Then she* [the daughter] *wrote back and* [presented new arguments] *and we got an answer straight away. And we got a respite place for* [the care recipient]. (Participant 8, aged 70–80, caring for a spouse)

In some cases, healthcare personnel were considered gatekeepers to additional support that participants believed their relative ought to receive. Some described how they utilized their social network to get around such gatekeepers. Participant 9 provided an example of this when explaining how they got their mother tested for dementia, despite her GP’s view that this was unnecessary:

*But then I happened to speak with this psychologist, because I happened to be at a course and he says this and that and, yes, he can do a dementia test. So I sent mum to that and on the basis of that, he believed that she needed a full assessment.* (Participant 9, aged 50-60, caring for a parent).

Other ways of getting around gatekeepers was to go above their heads and speak directly to managers, service leaders, or local politicians. When Participant 10 overheard home care nurses say that their sister [the care recipient] was denied a place at the nursing home, Participant 10 demanded a meeting with the manager of the nursing home and home care services. When the meeting took place, Participant 10 was able to provide detailed information about their sister’s situation directly to the decision maker:

*So I went home and picked up the phone to the manager and demanded a meeting…*[At the meeting] *I tell her* [the manager] *how I experience my sister, everything that has happened…She gets more and more shocked…Then she says “I haven’t heard any of this. No one told me this.” (Participant 10, aged 70–80, caring for a sibling)*

Participant 10 explained that as a result of the meeting, their sister was given a place at the nursing home. Other participants also gave examples of how they had convinced decision makers about the need for additional services. However, not all of them were as satisfied or as successful in their efforts as Participant 10. Some said that because they had not been able to convince service providers to increase their care provision, they were now exhausted and in despair, having provided extensive care for years.

#### Filing formal complaints

If former approaches had failed, and participants still had strong concerns about the quality of care, some chose to file formal written complaints. Participant 3 formally complained about their mother’s GP when he failed to provide a prescription for new medication suggested by hospital doctors, which meant the home care nurses were unable to administer what the family carer and the hospital physician thought was the best treatment. Participant 10, who was a professional nurse with specialist training in geriatric care, complained when their sister’s treatment for an acute infection was delayed, despite having expressed clearly to nursing home staff that they needed to start treatment immediately. Participant 2 said that they felt compelled to complain after their mother had suffered repeated injuries during respite stays at the local nursing home, which the staff could not explain. Participant 2 experienced that the healthcare personnel were polite and nice when approached about this, but as injuries continued to happen, they filed a complaint with the County Medical Officer:

*Participant 2: When she was at the respite care it was like “I wonder how she’ll look when I collect her this time. Is she yellow and blue?” Because she had cuts in several places.*

*KH: Fell several times, then?*

*Participant 2: Yes. And “nobody knows anything.” That is the weirdest thing about it. “Tell me, have you not [noticed]?” “Yes, but we haven’t reported”* [the staff replied]. *So we have filed with the County Medical Officer*. (Participant 2, aged 40–50, caring for a parent)

Filing complaints was described as a last resort, one which participants only initiated after careful consideration, because they feared it could damage their relationships with healthcare personnel.

### Weighing approaches

Examples of approaches from the assertive strategy were relatively uncommon in the narratives, compared to examples of approaches from the supportive and complementing strategy, but the stories that exemplified them were often presented as dramatic, negative experiences that were salient to the participants. Since adding pressure and momentum could create tension or conflicts, many expressed that they had to “choose their battles” and carefully weigh the risks of various approaches against other concerns.

Preserving good relationships with staff was one such concern. As explained above, Participant 5 worried about the care recipient’s food intake. Participant 5 also emphasized several times that they made a great effort to maintain a “good relationship” with the nursing home staff and was careful not to criticize healthcare personnel directly, despite worrying about the limited emotional support and the quality of medical care which the care-recipient received.

The opportunity cost of spending time and energy on “fights” and complaints rather than on other activities was mentioned by several participants. Participant 11 had been angered by what they perceived to be disrespectful treatment of the care recipient during discharge from the hospital. The care-recipient had been spoken to in what Participant 11 described as insensitive language with a harsh tone and had been given little time for explanations. Participant 11 had been advised by friends to file a complaint, but prioritized spending her time and energy on caring:

*The situation is as it is and there weren’t any beds* [in the hospital] *and all of that, but it is possible to convey that in an OK way. Not like* [makes spitting noise] *and like “off you go,”. Many people told me “you should report it.” But I don’t have the energy. I really have a few other things to concentrate on* [alluding to care tasks]. (Participant 11, aged 70–80, caring for a spouse)

As shown above, Participant 2 did at some point file a formal complaint with the County Medical Officer. However, when the office called some time later and asked if they wanted to pursue the case, their mother had moved to a nursing home and was no longer using the facility where these incidents had happened. For this reason, Participant 2 said that they now lacked both the incentive and the energy to continue the fight, even though they did considered it important:

*They called and asked if I wanted to pursue the case; that would’ve been June of last year. I said, “I can’t be bothered to pursue it, because now we’re not with the home care service anymore, she’s got a permanent place* [in a nursing home]. *But I hope you take this case further so that things can improve for others. But I can’t be bothered, because I’d be spending my time on nonsense.”* (Participant 2, aged 40–50, caring for a parent)

Participant 2 also said that the fear of being perceived as a “difficult” family carer was one reason why they sometimes let things go:

*Participant 2: But, you know, you can’t just keep complaining all the time.*

*KH: No? What will happen then? It sounds like you have things to complain about, really.*

*Participant 2: Yes, but no. I don’t know. I suppose it is that you live in a small place and they’ll think “oh, there they comes again”* [laughs]. *I don’t know.* (Participant 2, aged 40–50, caring for a parent)

In sum, participants weighed a range of concerns when considering how to involve themselves if they experienced that services were somehow insufficient. These concerns included potential consequences for the relationship with healthcare personnel and how best to spent their own time and energy. Some also worried about potential negative consequences for the care recipient, although this was more expressed as a “gut feeling” than illustrated with examples.

Many reflected on whether it might be unreasonable to place more demands on healthcare personnel who already had very busy work schedules. Participant 6 expressed this about advising others on how to combine making demands with showing respect for the personnel’s need to attend to many patients and many tasks:

*Well, don’t take things at face value. There are—God knows, there are many who do their best at those nursing homes. Don’t shout. Be critical, but don’t shout…I can speak very firmly sometimes. They need to understand that a spade is a spade. Yeah. But you’ve got to respect that they’re doing a lot of good, really, very many of them.* (Participant 6, aged 40–50, caring for a parent)

In deciding how best to involve themselves, participants like Participant 6 thus carefully considered potential costs and benefits for the person living with dementia, for themselves, and for healthcare personnel.

## Discussion

By interviewing family carers about how they got involved when they felt that services did not adequately meet the needs of their relatives living with dementia, we identified two involvement strategies. The first, and by far the most commonly described, largely involved complementing and supporting services. The strategy included different approaches for connecting disjointed services, completing information flows, and filling care gaps. In these approaches, participants portrayed themselves as “a hub in the wheel”. The second strategy was more assertive and included approaches to add power, momentum, leverage, or pressure to arguments or processes. To determine which approach to use in a given situation, participants weighed a range of considerations. We will address each of these issues before discussing how they influence the opportunity for good partnerships in care.

### The “hub in the wheel”: essential but often unnoticed

A hub links together the spokes of a wheel, carries some of the weight, and facilitates the spinning of the wheel—ideally with a minimum of friction. Such functions were a dominant theme in the narratives of how family carers involved themselves by linking disconnected services and improving communication between families and services. By taking on this role, participants aimed to facilitate seamless care and allow health services to run as smoothly as possible.

Descriptions of hub functions discussed common, everyday, and often rather mundane tasks, such as reminding hospital personnel to send the discharge note, ensuring that the correct information reached the right people, that the right equipment was in the right place, or that their relative was sufficiently dressed and fed. When the wheel functions well, little attention is paid to the hub. In similar ways, family carers’ contributions when using approaches associated with the “hub in the wheel” strategy, may be overlooked or taken for granted. While the literature on family involvement in care often emphasizes areas of tension or conflict [38, 63, 64] family carers’ efforts to complement, repair, or connect services also deserve recognition. We have shown previously that when family carers’ contributions go unrecognized, this can unintentionally lead to care gaps and to needs remaining invisible to health professionals [55]. It is therefore important that their involvement as “hubs” (which is well aligned with how policy describes their role) is recognized and acknowledged.

### Adding leverage by applying personal resources

A number of examples were given of how participants experienced that they needed to involve themselves in more assertive ways. The more assertive approaches added momentum or pressure which enforced improvements or changes, like getting the wheel rolling in the direction they believed was in the best interest of the person living with dementia. As mentioned above, participants’ narratives and experiences reflected an awareness of the power differentials between health professionals and family carers. This power imbalance is well described in the literature [65, 66], and for our participants, it often made it challenging to attempt to change healthcare personnel’s decisions or improve service provision. The approaches to add leverage and power to arguments or processes included visibly monitoring services, using relationships as leverage or to get around “gatekeepers”, and filing complaints. In doing so, participants drew on different types of personal resources at their disposal.

Several participants gave examples of how they involved people in their social networks to get around gatekeepers or to add weight to their arguments or positions, such as Participant 9 involving an acquaintance who was a psychologist. This and other examples showed how participants used their social capital (which refers to resources available through a person’s social networks) as part of their involvement strategies [33]. When Participant 7 questioned the legality of the nursing home’s routine of locking doors, they drew on her knowledge and experience from working in health care as a specialist nurse. This can be understood as drawing on their cultural capital (which refers to a person’s accumulated knowledge, expertise, or skills) [34]. Such use of personal resources was displayed by how some of the participants referred to professional standards and legal requirements and even by filing formal complaints.

Such personal resources and how they are utilized in health care interactions can be understood through the concept of Health Literacy (HL), which refers to a person’s capacity to obtain, process and use information about health and healthcare systems [35, 67]. As we have seen, family carers’ may capitalize on these resources turning them into leverage. Fundamental to the HL concept is that people differ in their capacity to successfully navigate healthcare systems [35]: those with higher levels of HL will be better able to find their way through and be proactive users of healthcare systems than those with lower levels [68]. As HL level might affect communication, relationships, approaches and involvement strategies, it is critical that healthcare personnel are aware that HL levels vary among the family carers with whom they interact.

Differences in HL levels have been found to be closely associated with socioeconomic differences [69], and HL has been used to, in part, explain inequality in health and health care utilization [70–73]. Since family carers are often involved in identifying, applying for, and interacting with health services on behalf of older adults living with dementia, family carers’ HL levels, along with their social and cultural capital, may therefore affect which services are received, and to some extent also care quality. This aspect of family carers’ personal resources and its implications for dementia care is still under-researched [74], and further studies are needed to establish whether differences in such resources impacts equal access to and outcomes of health services.

### Weighing conflicting concerns

The involvement approaches described in the interviews ranged from keeping healthcare personnel informed to filing formal complaints. In deciding how to proceed in any given situation, participants weighed conflicting concerns associated with the potential and actual costs and benefits of the various approaches.

Potential benefits of using approaches aligned with being the “hub in the wheel” include improved, more person-centered, seamless care to the person living with dementia. By almost unnoticeably involving themselves in care provision, they endeavored to build or maintain good relationships with healthcare personnel. However, using this strategy also had potential costs. Always being present to fill in or rectify information flows added responsibilities and was extremely time-consuming, and thus came with considerable personal costs. Since the supportive and complementing strategy was most commonly applied, such costs to carers may be extensive and could represent a threat to the resource they provide to services. Personal costs to the family carer are described in other studies [75, 76]—such as heavy carer burdens [77–80], negative health effects [81, 82], and reduced quality of life [83, 84]. It is well known that many family carers of older adults living with dementia spend significant amounts of time on caring duties and responsibilities [48].

At the other end of the spectrum, adding leverage and pressure could potentially improve care quality, obtain access to additional services, and remind healthcare personnel of their professional responsibilities. The assertive approaches had the potential costs of straining relationships with healthcare personnel or being perceived as a “difficult” family carer. Previous studies have shown how family carers worry about reprisals or retaliations when they act assertively [85], and many are worried it could affect the quality of care provided to their relative [86]. Such concerns indicate an awareness of the power differentials surrounding interactions between family carers and healthcare personnel [31, 87], which is recognized in the literature as a threat to real partnerships in care [31]. Asymmetric power relationship between family carers and physicians [29, 58] and other types of health care personnel [4, 31, 57] are recognized as impacting interactions in way ranging from deciding what information in relevant in a particular situation, to, making formal care decisions [57].

From the perspective of family carers, different approaches to involvement are associated with the costs that they weigh against the potential benefits. By being aware of this, and the context of asymmetric power relationships, healthcare personnel can acknowledge family carers’ efforts to contribute, which might help redress some of the imbalance in those relationships.

### Moving towards stronger partnerships in care

In a landscape of conflicting concerns, power differentials, and differences in personal resources or health literacy, family carers and healthcare personnel are expected to build partnerships in care. However, the way these partnerships are portrayed in policy documents often fails to take into account how family carers understand their responsibilities when their views and opinions differ from those of healthcare personnel, or when they perceive services to be insufficient or failing. As our findings show, family carers use different involvement strategies when they experience gaps or failures in services. One strategy is supportive or complementary, which is usually acceptable to and indeed welcomed by healthcare personnel, while the more assertive or challenging strategy might be unwelcome or result in strained relationships. Nonetheless, both strategies are central to how family carers understand their involvement and duty to enhance the quality of care; as such, it is important that healthcare personnel—who interact with them—and policymakers—who develop services for them—are aware of these strategies and acknowledge their role in improving care.

As mentioned above, a partnership should be based on trust, equality, mutuality, and shared goals [14]. Based on the accounts of our participants, it is clear that the conditions for good partnerships are not always present. The difficulties described by participants in trying to enforce changes or improvements, indicate that neither mutual understanding of the goal nor equal power to make decisions is always present. The power imbalance between healthcare personnel and family carers further indicates a lack of equality in the relationship. The fear of reprisals and the need to keep healthcare personnel on alert can be seen as signs of mistrust. Policies that expect good partnerships in care must take into account the dynamic between healthcare personnel and family carers supporting people living with dementia, as well as the strategies family carers use and the personal resources available to them in their efforts to build or maintain partnerships.

## Conclusion

Using in-depth interviews with family carers of older adults living with dementia, we found that participants used two main types of strategies in their efforts to participate in and influence formal service delivery: 1) being “the hub in the wheel”, and 2) getting the wheel rolling. The first type of strategy aimed to connect different services, tailor services to personal needs, and support and complement formal care delivery, maintaining good relationships with formal carers. The second strategy aimed to add more leverage to their arguments or processes in their effort to instigate improvements when they perceived services as insufficient and power imbalances made it difficult to influence decisions. Both strategies had potential costs and benefits, and many considerations were weighed when participants were choosing their approach.

Differences in personal resources—such as knowledge, motivation, and social and communication skills, which formed part of HL, may constitute differences in family carers’ abilities to access and interact with health services on behalf of older adults living with dementia. Further research is needed to investigate whether this might be a contributing factor to inequalities in health care utilization among older adults living with dementia. Policymakers, family carers, and healthcare personnel can benefit from increased awareness about different family carers’ perspectives, in order to establish stronger, more equal partnerships between formal and informal carers.

## Data Availability

The datasets generated and/or analyzed during the current study are available from the corresponding author on reasonable request.

## Abbreviations

KH: Kristin Häikiö, first author
MS: Mette Sagbakken, co-author
JR: Jorun Rugkåsa, last author
